# Vasorelaxation Induced by a New Naphthoquinone-Oxime is Mediated by NO-sGC-cGMP Pathway

**DOI:** 10.3390/molecules19079773

**Published:** 2014-07-08

**Authors:** Bruna P. V. Dantas, Thaís P. Ribeiro, Valéria L. Assis, Fabíola F. Furtado, Kívia S. Assis, Jeziane S. Alves, Tania M.S. Silva, Celso A. Camara, Maria S. França-Silva, Robson C. Veras, Isac A. Medeiros, Jacicarlos L. Alencar, Valdir A. Braga

**Affiliations:** 1 Biotechnology Center, Federal University of Paraíba, João Pessoa, PB 58.051-900, Brazil; E-Mails: brunapvd@gmail.com (B.P.V.D.); thaispribeiro@hotmail.com (T.P.R); val_farm@hotmail.com (V.L.A.); fabiola.fialho@gmail.com (F.F.F.); kivia_sales@hotmail.com (K.S.A.); francasilva@cbiotec.ufpb.br (M.S.F.-S.); robveras@msn.com (R.C.V.); isacmed@uol.com.br (I.A.M.); jacicarlos@ccm.ufpb.br (J.L.A.); 2 Molecular Sciences Department, Federal Rural University of Pernambuco, Recife, PE 52171-900, Brazil; E-Mails: quimicajeizi@yahoo.com.br (J.S.A.); taniasarmento@dcm.ufrpe.br (T.M.S.S.); ccelso@dcm.ufrpe.br (C.A.C.)

**Keywords:** blood pressure, mesenteric rings, nitric oxide, potassium channels, vasodilation, naphthoquinone oxime

## Abstract

It has been established that oximes cause endothelium-independent relaxation in blood vessels. In the present study, the cardiovascular effects of the new oxime 3-hydroxy-4–(hydroxyimino)-2-(3-methylbut-2-enylnaphtalen-1(4*H*)-one (**OximeS1**) derived from lapachol were evaluated. In normotensive rats, administration of **Oxime S1** (10, 15, 20 and 30 mg/Kg, i.v*.*) produced dose-dependent reduction in blood pressure. In isolated aorta and superior mesenteric artery rings, **Oxime S1** induced endothelium-independent and concentration-dependent relaxations (10^−8^ M to 10^−4^ M). In addition, **Oxime S1**-induced vasorelaxations were attenuated by hydroxocobalamin or methylene blue in aorta and by PTIO or ODQ in mesenteric artery rings, suggesting a role for the nitric oxide (NO) pathway. Additionally, **Oxime S1** (30 and 100 µM) significantly increased NO concentrations (13.9 ± 1.6 nM and 17.9 ± 4.1 nM, respectively) measured by nitric oxide microsensors. Furthermore, pre-contraction with KCl (80 mM) prevented **Oxime S1**-derived vasorelaxation in endothelium-denuded aortic rings. Of note, combined treatment with potassium channel inhibitors also reduced **Oxime S1**-mediated vasorelaxation suggesting a role for potassium channels, more precisely K_ir_, K_v_ and K_ATP_ channels. We observed the involvement of BK_Ca_ channels in **Oxime S1**-induced relaxation in mesenteric artery rings. In conclusion, these data suggest that the **Oxime S1** induces hypotension and vasorelaxation via NO pathway by activating soluble guanylate cyclase (sGC) and K^+^ channels.

## 1. Introduction

For many years, investigators worldwide have explored the mechanisms by which nitric oxide (NO) causes vasodilatation. Despite its structural simplicity, NO has a complex biochemistry, endowing the free radical with wide and varied biological actions [1,2]. The NO, which is synthesized from the amino acid L-arginine by the enzyme nitric oxide synthase (NOS), has emerged as a key molecule in the regulation of vasomotor tone and arterial pressure in both animals and humans [3]. In addition to the classical production of NO via NOS, the existence of NOS-independent pathways for NO production from exogenous substrates is of particular pharmaceutical interest [4,5,6].

In this regard, compounds presenting a R_2_C=NOH structural group can be metabolized by hemoproteins like horse radish peroxidase, rat liver microsomal cytochrome P450 (P450), hemoglobin, and catalase. This biotransformation process results in nitric oxide (NO) or NO-related vasorelaxant species formation in blood vessels that are independent of nitric oxide synthase activity [7,8,9]. Among the compounds containing the structural group (R=NOH) are the oximes, *N*-hydroxyguanidines, amidoximes and ketoximes [10,11,12].

**Figure 1:**
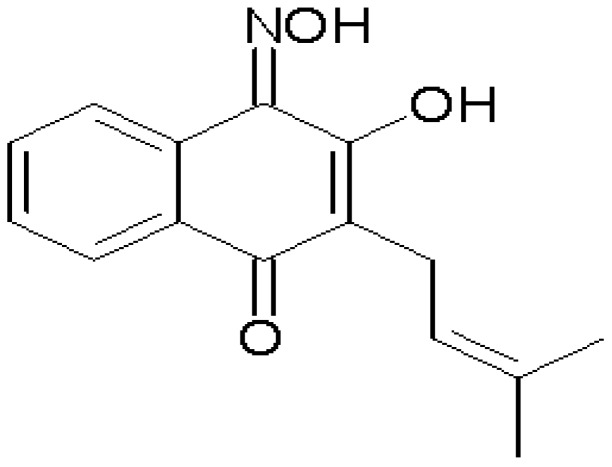
Structural formula of 3-hydroxy-4–(hydroxyimino)-2-(3-methylbut-2-enylnaphtalen-1(4*H*)-one (**Oxime S1**).

Pharmacological studies of different oximes (R_2_C=NOH) have shown that these compounds can induce vascular relaxation by different pathways. Previous studies aiming to evaluate the vascular effects induced by oximes were performed in arteries without endothelium suggesting the involvement of the NO-cGMP pathway [13,14]. The aim of the present study was to investigate the cardiovascular effects produced by a new synthetized oxime, the 3-hydroxy-4–(hydroxyimino)-2-(3-methylbut-2-enylnaphtalen-1(4*H*)-one (**Oxime S1**, Figure 1), which is a semi-natural naphthoquinone derivative of lapachol extracted from *Tabebuia avallanedae* Lor.Ex.Gris (*Bignoniaceae*), which formula is shown bellow.

## 2. Results and Discussion

### 2.1. Oxime S1 Reduces Blood Pressure in Conscious Rats

**Figure 2:**
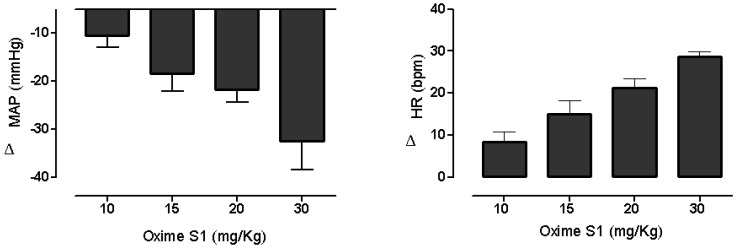
Changes in mean arterial pressure (MAP) and heart rate (HR) induced by the acute administration of increasing doses of **Oxime S1** (mg/kg, i.v.) in conscious normotensive rats. Values are expressed as mean ± SEM (*n* = 6).

Baseline values of mean arterial pressure and heart rate in rats (*n* = 6) were 107 ± 1 mmHg and 378 ± 5 bpm, respectively. Administration of **Oxime S1** (10, 15, 20 and 30 mg/Kg, i.v., randomly) induced dose-dependent hypotension (−10 ± 3, −18 ± 4, −22 ± 3 and −32 ± 6 mmHg, respectively) associated to increase in the heart rate (8 ± 3, 15 ± 3, 21 ± 4 and 25 ± 4 bpm) (Figure 2).

It has been reported that non-amino acid compounds sharing the R_2_C=NOH group can produce nitric oxide synthase-independent relaxation in endothelium-denuded aortic rings of rats [10,14,15,16]. In addition, the mechanisms underlying the effects of exogenous nitrovasodilators are predominantly mediated by cyclic guanosine monophosphate (cGMP), as a result of the activation of soluble guanylyl cyclase [17,18]. Cyclic GMP may cause vasodilation by the stimulation of cyclic nucleotide-gated channels [19]. Therefore, we suggest that the decrease in blood pressure elicited by **Oxime S1** under our experimental conditions could be due to NO release in vascular smooth muscle cells.

In order to investigate the direct effect of **Oxime S1** on the vasculature, we performed experiments in vascular preparations. Whereas compounds that release NO exert major effects on conductance vessels, we investigated the effect of the **Oxime S1** on aorta isolated from rat. In addition, due to the fact of resistance arteries play important role in determining baseline blood pressure, we also evaluated the effect of the compound on superior mesenteric arteries rings.

### 2.2. Oxime S1 Produces Endothelium-Independent Relaxations in Both Aorta and Superior Mesenteric Artery Rings

**Figure 3:**
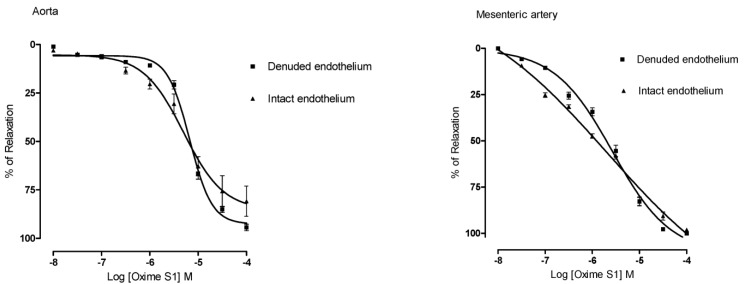
Concentration-response curves showing the relaxant effect of Oxime S1 (10^−8^ to 10^−4^ M) in aorta and mesenteric artery rings with intact endothelium (▲) and denuded endothelium (▀). The response is expressed as percentage of relaxation from the phenylephrine-induced contraction (100% means complete relaxation). Each data point and vertical bar represents the mean and the s.e.m. from 10 different experiments.

**Figure 4:**
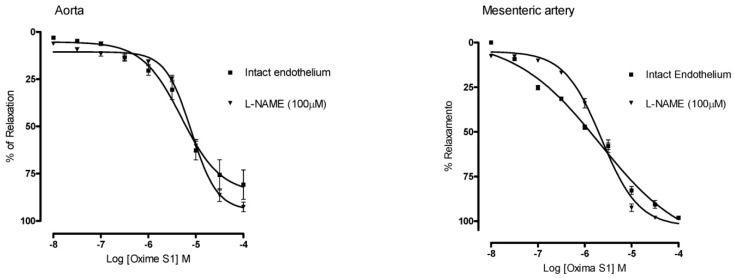
Concentration-response curves showing the relaxant effect of Oxime S1 (10^−8^ to 10^−4^ M) in aorta and mesenteric artery rings with intact endothelium (▀) and in the presence of L-NAME (▼). Each data point represents the mean and s.e.m. from 7 to 10 different experiments.

**Oxime S1** (10^−8^ to 10^−4^ M) produced concentration-dependent vasorelaxation in phenylephrine pre-contracted aorta and superior mesenteric artery rings isolated from rats in the presence of functional endothelium (E_max_ = 80% ± 15% in aorta; E_max_ = 98% ± 1% in mesenteric artery) (Figure 3). The relaxant response induced by the **Oxime S1** was not affected by endothelium removal (E_max_ = 94% ± 4% in aorta and E_max_ = 100% ± 1% in mesenteric artery, *p* < 0.001, as illustrated in Figure 3), suggesting that endothelium-derived products were not involved in the relaxation induced by **Oxime S1**. Furthermore, relaxations in aorta and mesenteric artery rings induced by **Oxime S1** were not affected by L-NAME (100 µM), an eNOS inhibitor, suggesting that endothelial NO synthase (eNOS) does not play a role in the relaxant effect induced by the compound (Figure 4).

### 2.3. Oxime S1 Produces Vasorelaxation via Activation of the NO-sGC-cGMP Pathway

**Figure 5:**
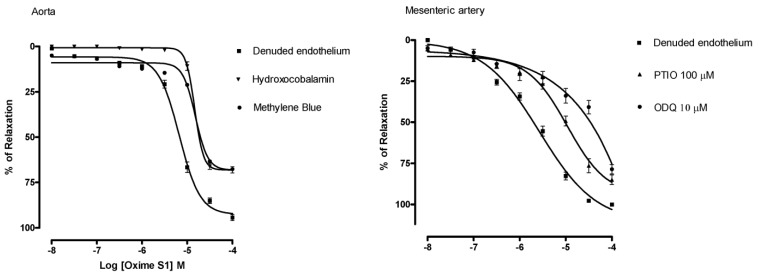
Concentration-response curves showing the relaxant effect induced by Oxime S1 (10^−8^ to 10^−4^ M) in aorta and mesenteric artery rings with denuded endothelium (▀) and in the presence of: Hydroxocobalamin (▼) or Methylene blue (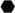) in aorta; PTIO (▲) or ODQ (●) in mesenteric artery. Each data point represents the mean and the s.e.m. from 7 different experiments.

Considering that oximes (compounds with a R_2_C=NOH group) can cause vasorelaxation by NO release, we investigated whether the **Oxime S1**-induced relaxation involves NO release. In fact, we have documented that oximes bearing a R_2_C=NOH group are able to release nitric oxide by using the NO indicator diaminofluorescein 4,5-diacetate [14]. The vasorelaxant responses induced by **Oxime S1** (10^−8^ to 10^−4^ M) in aorta rings were reduced by hydroxocobalamin (30 µM), an NO extracellular scavenger, when compared to control (E_max_ = 68% ± 4% *vs.* 94% ± 4%; and pD_2_ = 4.83 ± 0.04 *vs.* 5.20 ± 0.04, *p* < 0.05, respectively), and by methylene blue (10 µM), an inhibitor of sGC (E_max_ = 67% ± 5% *versus* 94% ± 4%; and pD_2_ = 4.85 ± 0,04 *versus* 5.2 ± 0.04, *p* < 0.05) (Figure 5).

In addition, treatment with PTIO (100 µM), an NO extra and intracellular scavenger, and ODQ (10 µM), an more selective inhibitor of sGC, attenuated the **Oxime S1**-induced vasorelaxation in superior mesenteric artery in a similar proportion to that observed in aorta rings (PTIO: E_max_ = 75% ± 6%; pD_2_ = 5.1 ± 0.07; ODQ: E_max_ = 68% ± 6%; pD_2_ = 4.8 ± 0,1, *p* < 0.05,) (Figure 5). These data suggest the involvement of the NO-sGC-cGMP pathway in the relaxant response induced by **Oxime S1**. In a similar way, Chalupský *et al.* and Veras *et al.* [13,14] demonstrated that the effects of several non-aromatic substituted oximes derivatives involve the NO-sGC-cGMP pathway.

### 2.4. Vasorelaxation Induced by Oxime S1 is also Mediated by Activation of K^+^ Channels

It is well known that NO plays an important role in the control of vascular tone and in the regulation of blood pressure through the activation of sGC in vascular smooth muscle leading to accumulation of cGMP [20] and subsequent cGMP-dependent protein kinase (PKG) activation [21]. The PKG phosphorylates several proteins such as the vascular smooth muscle K^+^ channels. Their activation results in cell hyperpolarization or repolarization due to K^+^ efflux, followed by subsequent closing of voltage-dependent Ca^2+^ channels, decreasing the intracellular level of Ca^2+^, leading to vasodilatation [22].

**Figure 6:**
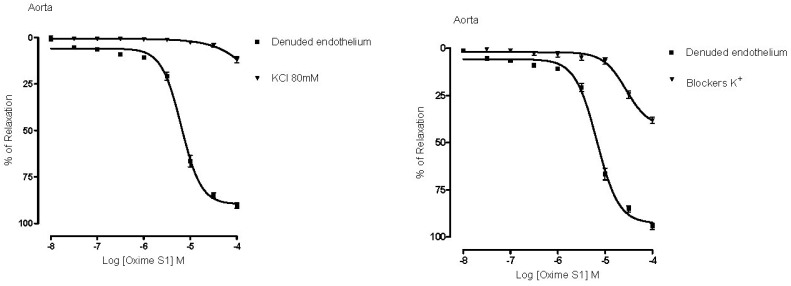
Concentration-response curves showing the relaxant effect induced by **Oxime S1** (10^−8^ to 10^−4^ M) in denuded endothelium from aorta in the presence of KCl 80 mM or potassium channels blockers. Values are expressed as mean ± s.e.m. Each data point represents the mean and the SEM from 7 different experiments.

In order to investigate the role of K^+^ channels in the vascular response induced by **Oxime S1**, we incubated aortic rings with combined K^+^ channels blockers (TEA, glibenclamide, 4-AP and BaCl_2_) and observed that **Oxime S1**-mediated vasorelaxation was blunted when compared to control (E_max_ = 94% ± 4% *vs.* 38% ± 3% and pD_2_ = 5.2 ± 0.04 *vs.* 4.6 ± 0.05, *p* < 0.05, respectively) (Figure 6).

In addition, membrane depolarization with KCl (80 mM) reduced the vasorelaxation produced by the oxime (from E_max_ = 94% ± 4% to 11% ± 4%; and pD_2_ = 5.2 ± 0.04 to 4.4 ± 0.1, *p* < 0.05) (Figure 6). The major consequence of increasing the extracellular K^+^ concentration (from 4 to 80 mM) is the reduction of the electrochemical gradient to K^+^ efflux. Under this experimental condition, substances that open K^+^ channels have their effect attenuated [22,23,24,25]. These data suggest the involvement of K^+^ channels in the response induced by **Oxime S1**.

**Figure 7:**
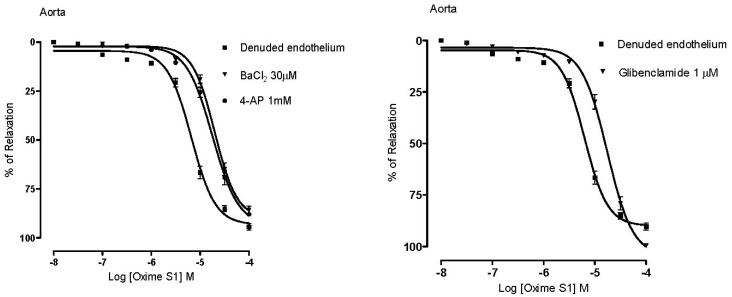
Concentration-response curves showing the relaxant effect induced by Oxime S1 (10^−8^ to 10^−4^ M) in aorta in the absence or presence of: BaCl_2_ (▼); 4-AP (●); or Glibenclamide (▼). Values are expressed as mean ± s.e.m. Each data point represents the mean and the s.e.m. from 7 different experiments.

In different series of experiments, pre-incubation with BaCl_2_ (30 µM), a selective inwardly-rectifying potassium channel (K_ir_) blocker; 4-AP (1 mM), a selective voltage-activated potassium channel (K_v_) blocker and GLIB (1 µM), a selective ATP-sensitive potassium channel (K_atp_) blocker, significantly attenuated the vasorelaxant response of **Oxime S1**. Dose-response curves were shifted to the right with pD_2_ values = 4.7 ± 0.03; 4.81 ± 0.03 and 4.82 ± 0.03, respectively (*p* < 0.05), suggesting the involvement of these channels in the **Oxime S1**-induced vasorelaxation (Figure 7).

**Figure 8:**
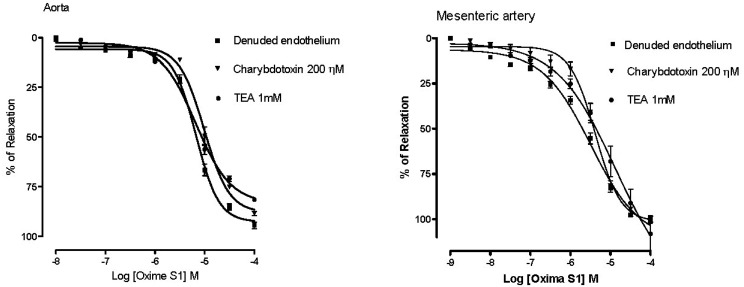
Concentration-response curves showing the relaxant effect induced by Oxime S1 (10^−8^ to 10^−4^ M) in aorta or mesenteric in absence or presence of: Charybdotoxin (▼) or TEA (●). Values are expressed as mean ± s.e.m. Each data point represents the mean and the SEM from 7 different experiments.

In contrast, when rings from aorta were incubated with TEA (1 mM) or ChTX (100 nM), large- and intermediate-conductance Ca^2+^-activated potassium channels (BK_Ca_) blockers, there were no changes in the values of E_max_ (81% ± 1% and 88% ± 1%, respectively) and pD_2_ (5.2 ± 0.03 and 5.0 ± 0.03, respectively) (Figure 8). Of note, the large conductance calcium activated K^+^ (BK_Ca_) channels is highly expressed in vascular smooth muscle cells and play an essential role in regulating resting membrane potential and, hence, vascular tone. Activation of BK_Ca_ channels in smooth muscle leads to efflux of K^+^ from the cell and causes hyperpolarization, which decreases the activity of voltage-gated L-type Ca^2+^ channels and subsequently leads to vasorelaxation. BK_Ca_ channel inhibition causes depolarization, increasing the activity of voltage gated L-type Ca^2+^ channels and subsequently leads to vasoconstriction. Interesting, the pre-treatment of mesenteric artery rings with TEA and ChTX attenuated the vasorelaxant response induced by oxime S1, demonstrated by the significant reduction in pD_2_ (5.3 ± 0.09 and 5.4 ± 0.05, respectively, *p* < 0.05) (Figure 8), suggesting that the action of oxime S1on K^+^ channels can be different according to the vascular bed evaluated.

### 2.5. Oxime S1 Releases Nitric Oxide When in Solution Acting as a Nitric Oxide Donor

**Figure 9:**
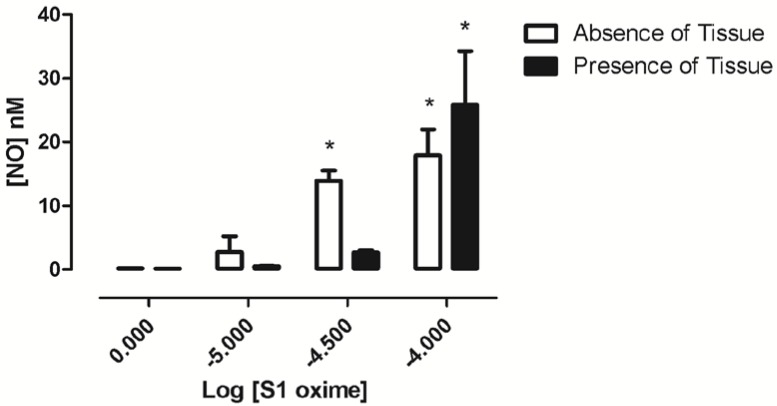
Effect of **Oxime S1** on NO concentrations. * means *P* < 0.05 when compared to baseline values prior to drug administration.

The use of electrochemical NO microsensors provides elegant and convenient approach to measure real-time NO release in biological samples. Therefore, in order to investigate whether **Oxime S1** could act as a nitric oxide donor, we measure the nitric oxide production when **Oxime S1** was added to the preparation with or without the rat superior mesenteric artery fragments. As shown in Figure 9, in the absence of tissue fragments, **Oxime S1** (30 and 100 µM) significantly increased NO concentrations while in solution (13.9 ± 1.6 nM; 17.9 ± 4.1 nM, respectively, * *p* < 0.05 *vs.* vehicle). In the presence of the tissue, **Oxime S1** (100 µM) significantly enhanced the concentration of NO in the preparation (25.8 ± 8.5 nM).

These data confirm that **Oxime S1** is able to release NO in a concentration-dependent manner, acting as a nitric oxide donor.

## 3. Experimental

### 3.1. Animals

Male Wistar rats (250–350 g) were used for all experiments. Animals were housed under controlled temperature (21 ± 1 °C) and lighting cycle (lights on: 06:00–18:00 h). In addition, rats had free access to food (Labina^®^, PURINA, Sao Paulo, Brazil) and tap water. Experimental protocols and procedures were approved by the Institutional Animal Care and Use Committee of the Federal University of Paraiba (Protocol 0610/05).

### 3.2. Drugs and Solutions

The drugs used were: Cremophor^®^, dimethyl sulphoxide (DMSO), (*R*)-(−)-phenylephrine hydrochloride, acetylcholine chloride, barium chloride (BaCl_2_), *N*^ω^-nitro-L-arginine methyl esther hydrochloride (L-NAME), L-arginine, 1*H*-[1,2,4]-oxadiazolo-[4,3-*a*]-quinoxalin-1-one (ODQ), 2-phenyl-4,4,5,5-tetramethylimidazoline-1-oxy-3-oxide (PTIO), tetraethylammonium chloride (TEA), 4- aminopyridine (4-AP), ketamine, xylazine, glibenclamide and charybdotoxin from Sigma-Aldrich (Saint Louis, MO, USA). Methylene blue and heparin sodium salt from Roche (Rio de Janeiro, RJ, Brazil) and hydroxocobalamin from Bristol-Myers Squibb (São Paulo, SP, Brazil).

The 3-hydroxy-4–(hydroxyimino)-2-(3-methylbut-2-enylnaphtalen-1(4*H*)-one) (**Oxime S1**, Figure 1) derivative of natural lapachol had its structure confirmed by infrared spectroscopy and mass spectrometry and was fully characterized by analytical and spectroscopic methods as earlier described [26]. **Oxime S1** was dissolved in a mixture of distilled water and Chremophor. The maximum concentration of Chremophor in the bath had never exceeded 0.01%. ODQ or glibenclamide was dissolved in DMSO. The composition of the Tyrode’s solution (in mM) was as follows: NaCl (158.3); KCl (4); CaCl_2_ (2); MgCl_2_ (1.05); NaH_2_PO_4_ (0.42); NaHCO_3_, (10) and glucose (5.6). The Kreb’s solution had the following composition (in mM): NaCl (118); NaHCO_3_ (25); KCl (4.6); MgSO_4_ (5.7); KH_2_PO_4_ (1.1); CaCl_2_ (2.5) and glucose (11).

### 3.3. Cardiovascular Parameters in Vivo

Arterial blood pressure and heart rate were recorded as previously described [27,28,29,30,31]. You jumped the numbers in between Briefly, under ketamine + xylazine anesthesia (70 and 10 mg/Kg, i.p.), rats were fitted with polyethylene catheters inserted into the abdominal aorta and caudal vena cava through left femoral artery and vein, respectively. Both catheters were tunneled subcutaneously, exteriorized and sutured at the dorsal surface of the neck. Twenty-four hours after surgical procedures, experiments were performed in non-anaesthetized rats. The arterial catheter was connected to a disposable pressure transducer (model TRA023, Harvard Apparatus, Holliston, MA, USA). The pressure transducer was connected to an amplifier-recorder (FE117, ADInstruments, Sydney, Australia) and to a computer equipped with an acquisition system (PowerLab, ADInstruments). Data were sampled at 1000 Hz using the software LabChart 7.0 (ADInstruments). For each cardiac cycle, the software calculated mean arterial pressure (MAP), and heart rate (HR). Changes in MAP and HR induced by S1 were compared among different doses (10, 15, 20 and 30 mg/kg, i.v.). After 30 min of baseline recordings, **Oxime S1** was administered randomly. Intravenous injections were performed every 15 min in order to allow cardiovascular parameters to return to baseline values.

### 3.4. Studies on Aortic and Mesenteric Artery Rings Isolated from Rats

Rats were euthanized by decapitation in guillotine. Thoracic aorta and superior mesenteric artery were harvested. In some cases, the vascular adventitial layer was separated from the media layer by microdissection. The intimal surface of the rings was rubbed to remove endothelial cells.

For experiments using the aortic rings (3–5 mm in length) and superior mesenteric artery rings (1–2 mm in length) were mounted in organ chambers filled with Krebs’ solution and Tyrode’s solution, respectively, maintained at 37 °C and pH 7.4, which was kept using carbogenic mixture (95% O_2_ and 5% CO_2_). Preparations were stabilized under a resting tension of 2 g for 1 h (aorta) and 0.75 g for 1 h (superior mesenteric artery). The presence of functional endothelium for aorta and superior mesenteric artery was assessed by the ability of ACh (1 µM and 10 µM, respectively) to induce more than 80% relaxation of vessels pre-contracted with Phe (1 µM and 10 µM, respectively). Less than 10% of relaxation to acetylcholine was taken as evidence that the vessel segments were functionally denuded of endothelium [29].

The force of contraction was isometrically recorded by a force transducer (Miobath-4, WPI, Sarasota, FL, USA) coupled to an amplifier-recorder (Miobath-4) and the rings were contracted with a single dose of phenylephrine or KCl (80 mM) and remained stable. **Oxime S1** (10^−8^ to 10^−4^ M) was cumulatively added to preparations until maximum responses were observed as indicated by a plateau (approximately 30 min). Inhibition was calculated by comparing the response elicited by **Oxime S1** before and after the inhibitors or antagonists were added to the preparation.

Based on our previous experiments, a standard incubation time of 30 min was adopted for the following inhibitors: L-NAME (100 µM); methylene blue (10 µM); ODQ (10 µM); hydroxocobalamin (30 µM); PTIO (100 µM); TEA (1 mM), 4-AP (1 mM), glibenclamide (1 μM), BaCl_2_ (30 μM) and charybdotoxin (200 nM).

### 3.5. Determination of Nitric Oxide Release Using NO Microsensors

Nitric oxide was measured according to the manufacturer’s instructions. Briefly, microsensors (ISO-NOP3005, WPI) were connected to an acquisition system (TBR 4100-Free Radical Analyzer, LABTRAX, WPI) and kept immersed for two hours in CuCl_2_ (0.1 M) to polarize and balance the amperage within a range of acceptability between 150–3500 pA. The microsensors were calibrated by decomposition of SNAP (S-nitroso-N-acetyl-d,l-penicillamine) by using a solution of CuCl_2_ (0.1 M) as active catalyst. The calibration curve was constructed by plotting the signal output *versus* the concentration of the SNAP added at that time. Changes in concentration of NO were determined in the absence and presence of rat superior mesenteric artery fragments (approximately 20 mm length), which were longitudinally cut and the luminal layer removed by mechanical rubbing. Then, the tissue was kept at 37 °C and aerated with gas mixture (95% O_2_: 5% CO_2_) in the NO measurement chamber (NOCHM-4, WPI), coupled to the microsensor (used in the calibration process) positioned nearest the tissue. Amperage variation were recorded before and after addition of increasing concentrations of **Oxime S1** (10, 30 and 100 μM). The concentration of NO was obtained from standard curves generated and analyzed in DataTrax-2 software (WPI). All measurements were performed in triplicate.

### 3.6. Statistical Analysis

Values were expressed as mean ± S.E.M. Statistical significance was determined by Student’s *t*-test or “*two-way*” ANOVA following Bonferroni’s post-test, when appropriate, using GraphPad Prism^®^ software, version 5.0 (GraphPad Software Inc., La Jolla, CA, USA). The maximal relaxation corresponds to the maximal effect (E_max_) of the highest concentration used. Pharmacological potency was determined by pD_2_ (negative logarithm of the concentration of a substance that induces 50% of maximal effect-log EC50). *p* < 0.05 were considered statistically significant.

## 4. Conclusions

In conclusion, using combined *in vivo*, *in vitro* and pharmacological approaches, we have demonstrated that the administration of 3-hydroxy-4–(hydroxyimino)-2-(3-methylbut-2-enylnaphtalen-1(4*H*)-one (**Oxime S1**) results in hypotension and NO pathway activation in smooth muscle cells with subsequent sGC and K^+^ channels activation. These findings shed some lights on the perspectives of using the new oximes for therapeutic purposes, especially for treating cardiovascular diseases such as hypertension.
